# Neurocognitive function and medical care utilization in Veterans treated for substance use disorder

**DOI:** 10.1186/s13011-024-00621-x

**Published:** 2024-08-30

**Authors:** James M. Bjork, Jarrod Reisweber, Paul B. Perrin, Paul E. Plonski, Clara E. Dismuke-Greer

**Affiliations:** 1 Mental Health Service, Central Virginia Veterans Affairs Health Care System, 1201 Broad Rock Blvd, Richmond, VA 23249 USA; 2 Virginia Commonwealth University, Richmond, VA USA; 3 University of Virginia, Charlottesville, VA USA; 4 Tufts University, Medford, MA USA; 5 VA Palo Alto Healthcare System, Palo Alto, CA USA

**Keywords:** Impulsivity, Addiction, Treatment, Health care, Cognition, Executive function, Veterans

## Abstract

**Background:**

Veterans with substance use disorder (SUD) are at high risk for cognitive problems due to neurotoxic effects of chronic drug and alcohol use coupled in many cases with histories of traumatic brain injury (TBI). These problems may in turn result in proneness to SUD relapse and reduced adherence to medical self-care regimens and therefore reliance on health care systems. However, the direct relationship between cognitive function and utilization of Veterans Health Administration (VHA) SUD and other VHA health care services has not been evaluated. We sought initial evidence as to whether neurocognitive performance relates to repeated health care engagement in Veterans as indexed by estimated VHA care costs.

**Methods:**

Neurocognitive performance in 76 Veterans being treated for SUD was assessed using CNS-Vital Signs, a commercial computerized cognitive testing battery, and related to histories of outpatient and inpatient/residential care costs as estimated by the VHA Health Economics Resource Center.

**Results:**

After controlling for age, an aggregate metric of overall neurocognitive performance (Neurocognition Index) correlated negatively with total VHA health care costs, particularly with SUD-related outpatient care costs but also with non-mental health-related care costs. Barratt Impulsiveness Scale scores also correlated positively with total VHA care costs.

**Conclusions:**

In Veterans receiving SUD care, higher impulsivity and lower cognitive performance were associated with greater health care utilization within the VHA system. This suggests that veterans with SUD who show lower neurocognitive performance are at greater risk for continued health problems that require healthcare engagement. Cognitive rehabilitation programs developed for brain injury and other neurological conditions could be tried in Veterans with SUD to improve their health outcomes.

**Supplementary Information:**

The online version contains supplementary material available at 10.1186/s13011-024-00621-x .

## Introduction

Medical recidivism, such as preventable hospital readmission, incurs substantial costs to private insurers, Medicare, the Veterans Health Administration (VHA), and other care systems [1, 2]. Not surprisingly, health services researchers working within these systems have attempted numerous interventions to improve patient adherence to prevent rehospitalizations, reduce length of stay, and lower costs [1, 2]. These interventions include improved pre-discharge patient education, better management by clinical staff of transitions in care, phone calls to discharged patients, home visits to check on discharged patients, and availability of patient hotlines [3].

Although rehospitalization or other potentially avoidable medical recidivism can result from systemic shortcomings in health system practices [4], medical recidivism can also stem from patient-level factors. These include low medication adherence [5], not attending outpatient appointments [6], and low adherence to exercise regimens and other elements of preventative self-care [7]. In many cases, logistical and physical barriers like transportation, poverty, and lack of culturally sensitive health care beyond a patient’s control preclude optimal medical adherence [8]. In other cases, however, low adherence to medication or exercise regimens and propensity for appointment no-shows may be associated with individual personality or cognitive features such as impulsivity [9] or from depression or impaired cognitive functioning [10].

One critical component of cognitive functioning germane to preventative self-care is executive function (EF), which encompasses working memory, set-shifting, and other mental functions that collectively subserve goal maintenance [11]. Low EF may hinder medical adherence by compromising a patient’s ability to attend to or to retain psychoeducation by providers or other patient education sessions or concepts. Subsequently, persons with low EF may forget or have low motivation to take self-care steps encouraged by providers. For example, low neurocognitive performance has been extensively linked to reduced self-care in individuals with diabetes [12], including in Veterans [13], and has been related to both lower frequency of blood sugar monitoring and of poorer foot care behavior [14]. In individuals with chronic obstructive pulmonary disease, fluid intelligence correlated with engagement in prescribed self-care behaviors such as vaccinations or use of supplemental oxygen, but crystallized intelligence did not [15]. In individuals with multiple sclerosis, low retention of verbally-presented information was associated with a greater likelihood of not attending one or more subsequent physical therapy sessions, and lower levels of working memory and processing speed were associated with not meeting rehabilitation goals [16]. One cognitive index particularly relevant to self-care is prospective memory, or “remembering to remember” to perform certain actions at future times such as taking medicine [17]. Successful prospective memory performance requires adequate EF, such as updating ability, in that behavioral responses need to change as a function of changes in the external environment, such as markers of time passage. Indeed, low levels of laboratory-measured prospective memory [18] and other EF [19] have been linked to reduced medication adherence.

Veterans with substance use disorder (SUD) may be particularly at risk for cognitive impairment. In addition to gradual reduction in cognitive function with normative adult aging, persons with SUD show reduced cognitive task performance relative to non-SUD controls in many if not most cross-sectional laboratory performance comparisons [20]. Polysubstance use disorder in particular is more common among individuals with lower performance on intellectual quotient (IQ) tests [21], although IQ test performance is highly correlated with poverty and education [22], which are also highly correlated with substance abuse [23]. Notably, reduced cognitive ability and especially sub-optimal decision making in laboratory tasks have been linked to poorer SUD treatment outcomes (reviewed in [24, 25]) with some evidence suggesting that patients with lower cognitive resources may benefit less from psychoeducation. Poor treatment outcomes would in turn lead to relapse and future new care encounters for SUD. Moreover, many Veterans experience persisting effects of traumatic brain injury (TBI) [26], where TBI may increase risk for SUD [27, 28]. Finally, Veterans who rely on VHA for health care tend to be of lower socioeconomic status than those seeking private health care services [29, 30], and resource scarcity itself has been linked to lower cognitive task performance [31], where this has been attributed to—among other systemic factors—economic worry constraining cognitive bandwidth [32].

Further, chronic SUD itself may result from certain EF impairments. The Addictions Neuroclinical Assessment (ANA) is a prevailing account for development and maintenance of SUD that attributes SUD to aberrant neurocircuit functioning that governs 1) incentive salience (of drug cues), 2) negative emotionality, and 3) EF [33]. Inhibitory control is typically considered one of the three primary components of EF (along with working memory and updating) [11] and may be the EF most germane to addiction [34]. Notably, inhibitory control entails both restraint of behavior as well as suppression of attentional bias. Low capacity for inhibitory control is thus thought to help maintain the cycle of addiction by not fully curtailing a person’s attentional bias toward drug-predictive cues or by not curtailing drug-seeking behavior in a similar mechanistic model of the “addiction cycle” [35] that focuses on brain and cognitive change across the sequence of binge, withdrawal, then preoccupation stages of a single substance-use bout.

Framed in terms of personality, reduced inhibitory control is thought to increase risk of each of substance use initiation, progression from recreational use to SUD, and risk of treatment dropout or SUD relapse by increasing *impulsivity* [34, 36, 37]. Impulsivity is a related multi-faceted construct that generally centers on “acting without thinking” or “living in the moment” with little regard for the future [38]. While impulsivity may be an adaptive strategy for obtaining unpredictable resources in chronically stressful or low-resource environments [39], laboratory task impulsivity shows a relationship with several unhealthy behaviors such as low medication adherence, risky sexual behavior, non-use of seatbelts, and texting while driving [reviewed in [9]]. Medication non-adherence [5], or low treatment session attendance [40] resulting from a general discounting of future bad outcomes in turn could result in hospital (re)-admissions. Elevated questionnaire (trait-based) impulsivity is also characteristic of SUD [41] and other mental disorders [42–44], and has been shown to partially mediate the linkage between PTSD and development of SUD [42] and increases risk of SUD relapse [45].

In sum, low neurocognitive performance and impulsivity are likely individual-level risk factors for SUD relapse and poor medical adherence generally in Veterans, that may in turn result in rehospitalization or other preventable high utilization of health care. In individuals with SUD, this would also include more likely re-engagements with SUD treatment due to relapse [24]. Despite the potential for low neurocognition to increase risk for non-adherence or medical recidivism, little is known about how neurocognitive performance relates to actual health care utilization. This could be evidenced by a relationship between lower neurocognitive performance and more intensive or frequent health care utilization, where estimated costs of care encounters could be used as a proxy for and quantification of care engagement. To our knowledge, the neurocognition-care utilization linkage has never been directly tested by blending objectively measured neurocognitive performance with administrative health care data. Demonstrating such linkages would provide impetus to develop psychoeducation programs that accommodate individuals with reduced neurocognitive functioning [46].

In the United States, the VHA system of care may be uniquely positioned for exploration of these neurocognition-care utilization relationships. Americans with both mental health diagnoses and low neurocognitive function are more likely to be unemployed or underemployed [47], and so lack access to employer-sponsored health insurance and care, despite passage of the Affordable Care Act [48]. In contrast, Veterans with limited resources are frequently afforded relatively increased access to health care by the VHA relative to access to private health systems, especially for treatment of health conditions determined to be partly or fully related to their military service [49]. Therefore, we posit that due to a greater access to care for service-connected health problems, an inverse relationship between neurocognitive performance and health care engagement is more likely to be detected in VHA data, and less likely to be confounded by individual differences in access to health care or by self-reports of health care utilization.

In a preliminary cross-sectional investigation, we administered a comprehensive computerized neurocognitive performance battery to Veterans who were visiting a large VHA medical center for SUD treatment, and related their neurocognitive performance and trait impulsivity to their nationwide VHA outpatient and inpatient/residential care costs, as determined by the longstanding algorithms of the VA Health Economics Resource Center (HERC) [50]. Our assessment centered on the omnibus performance scores and memory composite scores of the CNS-Vital Signs (CNS-VS) neurocognitive battery. This battery is composed of well-characterized subtests that capture working memory, set-shifting, attention, and other cognitive functions. In accordance with the linkages between lower neurocognitive performance and lower levels of medical adherence, we hypothesized that objective metrics of neurocognitive performance would correlate negatively with objective VHA health care costs in Veterans being treated for SUD. Demonstrating these linkages would indicate a need for greater efforts toward accommodating (if not rectifying [46]) reduced neurocognition in at-risk Veterans receiving medical care from the VHA, to improve their health outcomes.

## Methods

### Participants

Participants were Veterans (*N* = 76) who had participated in an unrelated study on cognitive features in different SUDs, for whom administrative cost data were available from HERC. Each was receiving inpatient or outpatient care in either a 28-day residential program, a 4-week intensive outpatient program, or 12-week, once-weekly outpatient group therapy at a VA medical center. Diagnoses of SUD per DSM-5 criteria were coded in the EMR by a program clinician based on a structured intake interview, as aided by both a pre-intake questionnaire and by urine toxicology results. A plurality (*N* = 33) of Veterans were classified as having poly-SUD based on meeting criteria for SUD-severe for more than one substance (typically including alcohol). Another *N* = 25 Veterans were being treated for alcohol use disorder (no other SUD moderate or severe), followed by *N* = 13 for a stimulant use disorder, *N* = 4 for opioid use disorder, and *N* = 1 for cannabis use disorder. The intake interview also asked Veterans about presence of TBI history, wherein *N* = 22 Veterans had self-reported histories of either “TBI” or “concussion” present in clinician notes.

### Neurobehavioral assessments

Impulsivity as a personality trait was assessed with the Barratt Impulsiveness Scale-11 [51], a 30-item multiple-choice questionnaire composed of items like: “I don’t pay attention” or “I say things without thinking.” Neurocognitive performance was assessed using the CNS-Vital Signs (CNS-VS) [52] computerized testing platform. The CNS-VS battery is a composite of several established neurocognitive tasks and has been extensively used to detect the effects of TBI and other neurological conditions. The CNS-VS has embedded validity (effort) checks. Participants whose subtask results were flagged as invalid were allowed up to two re-takes of those subtasks. Individuals who did not provide valid subtask re-takes after two attempts (*N* = 5) were excluded from the present dataset. The CNS-VS tasks were composed of the: Verbal Memory Test, Visual Memory Test, Finger Tapping Test, Symbol Digit Coding Test, Stroop Test, Shifting Attention Test, and Continuous Performance Test. Scores on these individual tasks collectively yielded an overall standardized (age- and sex-normed) Neurocognition Index (NCI) score as the primary metric of interest. Importantly, higher-order composites of several neurocognitive tasks typically show superior test–retest reliability compared to the reliability of individual cognitive tasks [53]. CNS-VS NCI test–retest reliability across a several-week interval (0.7) [54] compares favorably to composites from other computerized assessments. Due to a potential for memory problems to impede treatment adherence, we similarly analyzed the relationship between care costs and the CNS-VS Composite Memory scale score (derived from performance on the verbal and visual memory tests alone) and total care costs. The CNS-VS assessment took approximately 45 min, including rest breaks between tasks.

Because single-session neurocognitive assessments frequently do not correlate well with self-report trait questionnaire scores within-subject (especially regarding impulsivity) [55], we also administered the Barratt Impulsiveness Scale (BIS) [51] as a complementary metric of neurobehavioral characteristics that may reflect poor EF. To reduce comparisons, we utilized BIS total scores.

### Calculation of inpatient admissions and related costs

Inpatient admissions (which included residential care) across the nationwide VHA network for each Veteran in the study were obtained from the HERC Inpatient Database. Each record contained length of stay for each admission, and total costs for care for that admission from 2003 through mid-2022, using the approximations and algorithms of HERC, as described in [50, 56]. These costs are estimated by HERC using actual cost data from VHA facilities and estimates provided by Medicare. HERC inpatient costs include acute hospital care, inpatient non-acute care, and long-term care. Estimates of hospitalization costs are based on Medicare Severity Diagnosis Related Groups (MS-DRGs) and relative value units (RVUs) from Medicare. The MS-DRGs reflect the complexity of the patient in the weights assigned to each MS-DRG. The VHA RVUs reflect the effort of providers to treat a patient during an inpatient stay. Thus, HERC acute care cost estimates reflect the total cost of resources used in treating the patient. Inpatient non-acute care estimates are calculated for the cost of inpatient hospitalizations in rehabilitation, domiciliary, psychiatric, substance abuse, and intermediate medicine treatment units. To estimate the cost of non-acute inpatient care, HERC estimates the average cost of a day of stay and applies it to all days of stay, assuming that each day has the exact cost.

Outpatient visit costs in VA facilities are estimated by using the relative values of all Current Procedures and Terminology codes assigned to the visit in a VA facility. HERC uses the relative values from the Resource Based Relative Value System, a method that Medicare uses to reimburse providers for services provided to Medicare patients. HERC assigns every VA visit to one of 12 different categories of outpatient care: Outpatient Medicine, Outpatient Dialysis, Outpatient Ancillary Services, Outpatient Rehabilitation, Outpatient Diagnostics Services, Outpatient Prosthetics, Outpatient Surgery, Outpatient Psychiatry, Outpatient Substance Abuse Treatment, Outpatient Dental, Outpatient Adult Daycare and Home Care. HERC converts the relative value to a VA cost estimate for each category. HERC assumes that the resources used to provide VA outpatient care are proportionate to the relative values assigned in the Medicare reimbursement.

Hereafter, “hospitalization” denotes admission to either residential or inpatient VHA facilities. Raw costs of each outpatient encounter or individual admission/stay provided by HERC were first adjusted to 2022 dollars using annual inflation rates derived from the Consumer Price Index, then were summed for each participant, along with tallies of cumulative length of stay (in days) for all inpatient/residential admissions. Inpatient/residential values were provided by HERC were already classified by VHA-defined care categories, which included “substance abuse” and “psych.” We inferred “substance abuse” costs as treatment for SUD per DSM-5 criteria, and “psych” costs as treatment for PTSD, depression, or other non-SUD mental health (MH) disorders. Each outpatient encounter cost record was manually bifurcated into SUD-related versus non-SUD-related costs based on clinic stop code identifiers. Due to nuances of this clinical coding system, however, it was not feasible to further delineate non-SUD outpatient costs between MH-related and non-MH related outpatient costs. We summed the costs of these five inpatient and outpatient categories to calculate total VHA facility care costs.

Statistical analysis

### Statistical analysis

We first examined simple bivariate relationships between each of CNS-VS NCI scores, CNS-VS Composite Memory Scores, and BIS scores and total VHA facility health care costs. These were Spearman rank-order correlations to minimize effects of outlier care cost values. Three multiple regression analyses were also performed in parallel to examine the relationship between each of BIS scores, CNS-VS NCI scores, and Composite Memory subscale scores (as independent variables in the respective analyses) and total VHA facility health care costs (as the dependent variable) after controlling for age (independent variable). This was to account for the strong positive relationship between age and health problems and health care utilization generally [57] and to account for individual differences in the greater possible span of years when VHA care was available to a participant. For these regression analyses, inpatient (non-SUD) MH costs and inpatient Other costs were ln-transformed to correct the skewness (> 2.0) attributable to some very high-engagement Veterans. These six neurobehavioral-cost relationships were then Bonferroni-corrected. Finally, to provide a basic impression of the specificity of care costs for SUD versus other categories of VHA care, care cost categories were Spearman intercorrelated within-patient.

## Results

### Sample characteristics

Health care utilization statistics, including tallies of individual admissions and cumulative days of inpatient/residential care across admissions are shown in Table 1. All five HERC-estimated facility care category costs intercorrelated within-subject. For example, each Veteran’s outpatient SUD care costs not only correlated highly with their inpatient/residential SUD care costs ((Spearman) *r* = 0.710, *P* < 0.001), but also with their inpatient MH costs (*r* = 0.507, *P* < 0.001) and their inpatient Other costs (*r* = 0.702, *P* < 0.001). Similarly, *non-*SUD outpatient costs also correlated with each of inpatient MH costs (*r* = 0.475, *P* < 0.001) and inpatient Other costs (*r* = 0.529, *P* < 0.001). Finally, Inpatient SUD (residential care) costs correlated with each of inpatient MH costs (*r* = 0.467, *P* < 0.001) and inpatient Other costs (*r* = 0.455, *P* < 0.001). With regard to inter-relationships between non-cost metrics, BIS scores correlated negatively with age (*r* = -0.410, *P* < 0.001), but CNS-VS NCI scores did not after multiple-comparisons correction (*r* = -0.226, *n.s.*). BIS total scores and CNS-VS NCI scores did not intercorrelate (|r|< 0.05, n.s.). There were no significant differences in BIS scores or CNS-VS metrics between those Veterans who did versus did not endorse a history of one or more TBIs (all *P* > 0.30).

**Table 1:** Participant characteristics

	**Participant (n = 76) demographics**				
	Sex:		68		
		Female	8		
		Mean	SD	Median	Range
	Age:	50.7	11.3	52.5	28–68
	Estimated VHA care costs^a^	Mean	SD	Median	Range
	SUD residential/inpatient costs:	$24,557.56	$26,913.92	$21,064.47	$0-$105,571.35
	SUD outpatient costs:	$18,855.89	$21,217.31	$12,123.10	$77.54-$98,339.66
	Other MH residential/inpatient costs:	$40,659.00	$96,177.48	$0.00	$0-$575,854.71
	Other residential/inpatient costs:	$70,334.40	$119,845.00	$10,326.39	$0-$611,104.44
	Non-SUD outpatient costs:	$94,989.19	$89,905.08	$54,264.88	$2,027.88-$361,765.53
One or more SUD admissions: *N* = 46 (60.5%)	Number of SUD admissions^b^:	1.6	1.0	1	1–5
	SUD length-of-stays (d)^b^:	42.9	25.6	28	4–132
One or more other MH admissions: *N* = 37 (48.7%)	Number other MH admissions^b^:	4.8	5.9	2	1–29
	Other MH length-of-stays (d)^b^:	42.8	70.7	13	1–336
One or more Other admissions: *N* = 45 (59.2%)	Number Other admissions^b^:	6.3	9.3	3	1–52
	Other length-of-stays (d)^b^:	122.8	171.2	32	1–758

^a^Inflation-adjusted to 2022 dollars

^b^Among participants with one or more admissions of that type

### Neurobehavioral metrics and VHA care costs

**Fig. 1:**
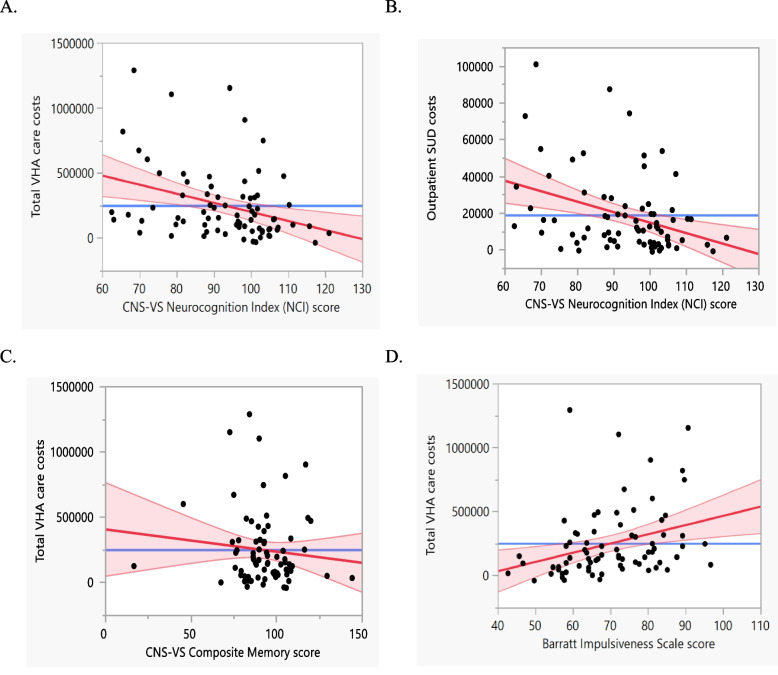
Shown are leverage plots stemming from multiple regression analyses that illustrate the relationships between Veterans Health Administration (VHA) estimated facility health care costs and neurobehavioral variables, after controlling for Veteran age. Neurocognition Index (NCI) scores from the CNS Vital-Signs (CNS-VS) battery related to total estimated VHA health care costs (Panel **A**; Std Beta = -.338, *P* = .003) and in particular SUD outpatient care costs (Panel **B**; Std Beta = -.369, *P* = .001. However, CNS-VS Composite Memory scale scores did not significantly relate to total VHA Care Costs (Panel **C**; Std Beta = -.103, n.s.). Barratt Impulsiveness Scale (BIS) total scores showed a positive relationship with Total VHA care costs (Std Beta = .341, *P* = .006)

In bivariate (Spearman) correlation, CNS-VS NCI scores correlated with total VHA facility care costs (*r* = -0.391, *P* < 0.001), indicating that lower overall neurocognitive performance was linked to greater engagement in VHA care in terms of costs. For example, NCI scores were significantly lower in patients who had one or more residential admissions for SUD (*N* = 46, mean 89.4) compared to those whose SUD care was outpatient only (*N* = 30, mean 98.6) (*t*[74] = 3.06, *P* = 0.003). However, the Spearman correlation between BIS scores and total VHA care costs (*r* = -0.245, *P* = 0.035) did not survive correction. CNS-VS Composite Memory scores also did not correlate with total VHA care costs (*r* = -0.142, *n.s*). After controlling for age in multiple regression, total VHA care costs still showed an inverse relationship with each of CNS-VS NCI scores (Std Beta = -0.338, *P* = 0.003) (See leverage plots in Fig. 1). In the multiple regression with BIS total scores (trait impulsivity) as predictor, higher BIS scores (Std Beta = 0.341, *P* = 0.006) and older age (Std Beta = 0.345, *P* = 0.006) each independently related to higher total care costs. Conversely, there was no significant relationship between CNS-VS Composite Memory subscale scores and total VHA care costs (Std Beta = -0.07, n.s.).

To provide insight into which specific cost categories may have driven the total care cost relationships described above, we performed similar exploratory analyses of how CNS-VS NCI scores and BIS scores related to each of the five specific category costs (as the dependent variable). These standardized Beta values (with uncorrected *P* values) are shown in Table 2. After controlling for age, CNS-VS NCI scores showed the strongest negative relationship with SUD outpatient care costs (Std Beta = -0.369, *P* = 0.001; Fig. 1, part B), and BIS scores showed the strongest positive relationship with SUD inpatient care costs, specifically (Std Beta = 0.506, *P* < 0.001).

**Table 2:** Relationships between cognition and care costs

	**Bivariate Spearman**		**Controlling for age**^a^:	
	**CNS-VS NCI**	**BIS Total**	**CNS-VS NCI**	**BIS Total**
Inpatient SUD	**-.329**	**.306**	-0.19	**0.506**
Inpatient MH	**-.332**	**.370**	-.070^a^	.295^a^
Inpatient Other	**-.373**	.146	-.234^a^	.117^a^
Outpatient SUD	**-.370**	**.297**	**-0.369**	**0.378**
Outpatient non-SUD	**-.284**	.220	**-0.282**	0.223

Bold denotes significant at *P* < .05 uncorrected

^a^Inpatient MH and Inpatient Other costs were ln-transformed

We also conducted exploratory analyses of which specific cognitive domains of the CNS-VS and which subscales of the BIS-11 related most closely to (total) costs of VHA care. After controlling for age, individual differences in processing and psychomotor speed, cognitive flexibility and the estimate of EF were most related to total VHA care costs (uncorrected relationships are shown in Supplemental Table 1). Each BIS-11 subscale (attention, motor, non-planning) showed a similar significant (but uncorrected) relationship with total VHA care costs.

## Discussion

We sought evidence as to whether either neurocognitive performance or trait impulsivity in a population at risk for lower cognitive functioning shows a relationship with health care costs using the VHA as a model system of care and by leveraging the established methods of cost analysis of the VA Health Economics Resource Center (HERC). In line with our hypotheses, we found in a sample of Veterans currently in SUD therapy that lower overall neurocognitive performance (but not working memory performance specifically) was positively associated with total and category-specific VHA facility care costs. In addition, once the normative decreases in questionnaire-measured impulsivity with aging across adulthood [58] were accounted for in multiple regression, trait impulsivity showed a positive relationship with VHA care costs.

Our findings are in accord with previous findings in another population of Veterans in SUD treatment, in whom HERC-estimated VHA care costs related to a different measure of trait impulsivity, and this relationship was also not specific to SUD care costs [59]. Here we replicated those findings and extend them—for the first time in the known research literature—to individual differences in neurocognitive performance. Interestingly, both trait impulsivity (BIS scores) and neurocognitive performance (CNS-VS NCI) correlated with care costs, even though the two metrics did not directly correlate with each other. The lack of a correlation between questionnaire and laboratory behavioral measures reflects many previous findings [55, 60, 61]. There may be two different pathways wherein facets of impulsivity [38] increase health care costs. An impulsivity facet such as myopic decision-making relies on valuation processes operating at longer time scales and could impact health care *planning* by placing low subjective value on health of future self by not taking the time to call to fill a prescription, to schedule a care appointment or to arrange transportation to care. A more motoric or EF performance-related impulsivity facet would operate on shorter time scales, such as failure to remember an impending appointment or failure to terminate consumption of a psychoactive substance or an unhealthy snack after initially indulging.

One potential account for the cognition-cost relationships is that they may stem in part from more severe and chronic SUD resulting in both: 1) more relapse-driven (re-)admissions and related costs as well as 2) more acquired brain toxicological insult with more years of harder substance use. Indeed, this investigation was motivated by a concern that due to chronic, heavy substance use, Veterans with SUD may be at risk for EF and other cognitive impairment [62] that could impede medication adherence, appointment attendance, attention to and recall of provider recommendations, and engagement in other preventative behaviors [10]. To this point, we note that CNS-VS scores also inversely related to the number of actual non-SUD/non-MH inpatient admissions (data not shown). We also reasoned that reduced EF could be especially possible in Veterans who sustained a TBI while intoxicated [63] or from combat zone deployment or other causes [64]. However, we found that there were no significant differences in cognitive performance between Veterans who reported a history of one or more TBIs versus those who did not. This similarity in scores may have stemmed from the low severity of TBIs described when such details were available. We caution, however, that the assessment of TBI history was a cursory self-report question, with no utilization of a more structured TBI assessment instrument. However, we caution that this causality cannot be confidently determined from the data at hand in that Veterans typically alternated between receiving SUD and non-SUD related care over time. This could in turn reduce Veteran health, requiring re-hospitalization and triggering related—often substantial—costs [1, 2]. 

Interestingly, estimated care costs in the different categories significantly intercorrelated within-patient. The correlations between SUD care costs and non-SUD mental health care costs may stem from individual differences in overall (non-specific) MH burden. Notably, examination of comorbid symptom patterns in large-scale longitudinal study supports the concept of a unitary, non-specific latent factor that indexes an overall MH symptom severity burden [65]. By extension, the same Veterans who have a severe SUD may also tend to have severe comorbid mood symptomatology. Correlations between SUD costs and non-MH “other” costs may have resulted from the significant medical comorbidities that can stem from chronic SUD [66]. Finally, these intercorrelations may have stemmed in part from individual differences in willingness to seek and utilize VHA care in general, given the same severity of symptom burden [67].

We also hypothesized that memory function in particular might be related to care costs by way of hindering the encoding of psychoeducational materials or the recall of provider instruction, or by increased propensity to forget to take medication or attend a scheduled care appointment, resulting in recurrence of medical conditions. Because the CNS-VS includes neither a prospective memory task, nor a working memory task, nor does it probe long-term episodic memory retention, we examined whether a composite of visual and verbal memory performance as a proxy metric related to total VHA care costs. In light of findings that episodic and working memory performance can not only correlate within-subject, but may be interdependent [68, 69], we reasoned that composite memory performance on the CNS-VS could serve as a proxy for other memory. However, the CNS-VS Composite Memory score showed no significant relationship with care costs. It is likely that verbal or visual memory itself correlates only modestly with episodic memory (and especially prospective memory) and so is not particularly germane to medical adherence.

Although the median standardized CNS-VS NCI score of our sample was 95.5 and suggestive of unimpaired neurocognitive performance relative to age- and sex-matched population norms, Fig. 1 leverage plots illustrate that whereas participants with NCI scores above this median typically incurred lower care costs, participants who scored below the normal range (i.e. ≤ 90) tended to incur higher care costs. We caution that the associations herein should not be construed as a justification for stigmatizing Veterans who have greater chronic health care needs and or neurocognitive limitations stemming from chronic SUD, TBI, or other sources. Rather we hope these findings will raise awareness of the possibility that presence of lower neurocognition in Veterans also confers risk for reduced health outcomes, with the goal of remediating neurocognitive impairments to improve health outcomes.

Fortunately, several cognitive rehabilitation programs have been developed that could improve cognitive and occupational functioning in these Veterans, such as those developed for persons with TBI [70]. For example, VA researchers have developed the CogSMART 12-week manualized therapy for individuals with TBI to improve prospective memory, attention, and other functions with weekly training of mental skill development [71]. These include memory retrieval strategies, conversation monitoring skills (social function), and other compensatory workarounds. In addition to improving prospective memory, this intervention has been shown to reduce self-reported cognitive problems in daily living over standard of care and employment supports in a comorbid PTSD + TBI Veteran population [46]. Although CogSMART and similar programs were originally motivated by post-concussive cognitive symptoms, their effectiveness could also be evaluated in an SUD treatment population. There is some preliminary evidence of beneficial effects of cognitive rehabilitation in SUD treatment populations [72], where for example this training has improved EF as well as quality of life [73].

### Study limitations

As a preliminary exploration of how neurocognitive performance relates to health care utilization, this report should be considered in light of several limitations. First, it was not feasible to reconstruct incidence or ratios of missed appointments themselves, or to quantify degree of medication and other treatment non-adherence from the data at hand. Therefore, relationships between reduced neurocognitive performance and care costs cannot be confidently attributed to low treatment adherence specifically. Future larger investigations could utilize more complex automated data-mining tools to glean these markers (e.g., rates of prescribed medication fills, level of income, etc.). Second, although the CNS-VS battery captures several cognitive domains, it is a relatively brief assessment compared to clinical neuropsychological assessments and does not include a conventional working memory task that requires retention and invocation of information on short time scales.

As noted above, other individual difference factors, such as overall addiction severity and substance effects on the brain, or systemic influences, such as chronic stressors, poverty, and unpredictable resource allocation, could account for these relationships. Importantly, poverty, reduced cognitive performance, SUD, and health behaviors are all interrelated [8], so unmeasured systemic variables (e.g., discrimination, chronic stressors, unpredictable resources, etc.) may also be influencing these relations. Relatedly, in light of how the population of Veterans who opt to receive health care at the VA is heavily skewed toward minoritized or otherwise disadvantaged Veterans (who also tend to have more severe mental health symptomatology and other health concerns [30]), these cost-cognition relationships may not generalize to Veterans whose health care is covered by private health insurance. Moreover, these findings may not generalize to other patient populations treated at VHA facilities, such as internal medicine or orthopedics. We note, however, that CNS-VS scores also inversely related to costs for general medical care that pertained neither to SUD treatment nor to other mental health disorders.

In addition, the sample size was not large enough to examine other potential moderating factors like sex or primary substance of disordered use on the neurocognition-care relationship. Third, although our key analyses relied on a composite cognitive performance metric (CNS-VS NCI) instead of a single neurocognitive task to improve reliability, the CNS-VS was only administered once, and so was subject to chance within-subject variation in sleep, pain or other transient factors, such that neurocognitive performance measures are generally more unreliable than self-report trait measures [55], such as with respect to predicting real world psychosocial outcomes [74]. Also, the frequent alternation over time between inpatient vs outpatient and SUD vs non-SUD VHA care encounters in nearly all participants precludes definitive evidence of causality wherein SUD care episodes suggestive of heavy substance use is uniquely predictive of future medical care costs. Finally, we had no information on care costs each participant incurred outside of VHA. Nevertheless, these data illustrate for the first time how lower neurocognitive performance may not only relate to reduced health behaviors directly [12], including in Veterans [13], but can also show a relationship with general care costs to the system.

## Conclusions and future directions

In conclusion, this study demonstrated for the first time a direct relationship between individual differences in neurocognitive performance and costs to a health care system. We also replicated a previous finding showing a relationship between self-reported trait impulsivity and care costs [59]. Future research with larger samples could examine moderators of these relationships such as presence of logistical or systemic barriers to medical adherence, and could administer more specialized cognitive probes such as prospective memory performance [75] that are likely to be more germane to psychoeducational retention and medication and other treatment adherence. Such findings may identify Veterans who could disproportionately benefit from other supports for medical adherence to improve their health outcomes.

### Supplementary Information

Supplementary Material 1.

## Data Availability

The datasets used and/or analyzed during the current study are available from the corresponding author on reasonable request.

## Abbreviations

ANA: Addictions Neuroclinical Assessment
BIS: Barratt Impulsiveness Scale
CNS-VS: CNS Vital Signs
DSM-5: Diagnostic and Statistical Manual for Mental Disorders, 5th Edition
EF: Executive Function
EMR: Electronic Medical Records
HERC: Health Economics Resource Center
IQ: Intelligence Quotient
MH: Mental Health (Service)
MS-DRG: Medicare Severity Diagnosis Related Group
NCI: Neurocognition Index
PTSD: Post-Traumatic Stress Disorder
RVU: Relative Value Units
SUD: Substance Use Disorder
TBI: Traumatic Brain Injury
VA: (Department of) Veterans Affairs
VHA: Veterans Health Administration

## Data Availability

An anonymized datasets as analyzed during the current study is available from the corresponding author on reasonable request.
